# Non-Invasive Ambient Intelligence in Real Life: Dealing with Noisy Patterns to Help Older People

**DOI:** 10.3390/s19143113

**Published:** 2019-07-14

**Authors:** Miguel Ángel Antón, Joaquín Ordieres-Meré, Unai Saralegui, Shengjing Sun

**Affiliations:** 1 Tecnalia, Parque Científico y Tecnológico de Gipuzkoa, Mikeletegi Pasealekua, 2. 20009 San Sebastián, Spain; 2 Escuela Técnica Superior de Ingenieros Industrales, Universidad Politécnica de Madrid, José Gutiérrez Abascal 2, 28006 Madrid, Spain; 3 i3-crg, École Politechnique, Route de Saclay, 91128 Palaiseau, France

**Keywords:** smart building, IoT, machine learning, ambient intelligence, ambient assisted living

## Abstract

This paper aims to contribute to the field of ambient intelligence from the perspective of real environments, where noise levels in datasets are significant, by showing how machine learning techniques can contribute to the knowledge creation, by promoting software sensors. The created knowledge can be actionable to develop features helping to deal with problems related to minimally labelled datasets. A case study is presented and analysed, looking to infer high-level rules, which can help to anticipate abnormal activities, and potential benefits of the integration of these technologies are discussed in this context. The contribution also aims to analyse the usage of the models for the transfer of knowledge when different sensors with different settings contribute to the noise levels. Finally, based on the authors’ experience, a framework proposal for creating valuable and aggregated knowledge is depicted.

## 1. Introduction

It has been largely demonstrated that huge improvements in human health and the increased attention being paid to the impacts of different factors (e.g., food and habits (smoking, sports, etc.)) on quality of life are resulting in the increased longevity of humans. According to a United Nations report [1], it is expected that there will be more than one billion people aged over 65 years by 2030, which is equivalent to one in eight people on Earth. Significantly, the most rapid increases in the 65-and-older population are occurring in developing countries, which will see a jump of 140% by 2030. This effect will dramatically impact the required infrastructures (e.g., homes). Therefore, it will be necessary to possess smarter ways to evaluate people’s behaviour and needs. There are different approaches for handling this, starting from ambient intelligence (AmI), which refers to electronic environments that are sensitive and responsive to the presence of people. It is possible to identify “ambient assisted living” (AAL) aiming to help individuals to continue to carry out their daily activities in their homes, for as long as possible and with the least amount of assistance.

Although there are research works aimed at tracking the movements of individuals with high precision, in order to gain knowledge regarding how the interactions develop for different tasks, as a wide variety of Internet of Things (IoT) devices are used [2,3], this paper aims to discuss how to exploit the advantages of the knowledge gathered in much more real contexts with non-intrusive sensors. The ambition of this work is to accept high levels of noise in the dataset, which is not very rich in terms of detail, and to try to derive useful behavioural rules to be applied in the same or other environments. The noise arises because of the usage on general non-intrusive sensors in real ambiences, where many uncontrolled factors such as not properly closed room door, not foreseen requests, etc., can affect the measures from those sensors. As far as those features are not directly related to the outcome of the model, they can be seen as noise for the learning process. Our major motivation is the fear that most users report the unwanted intrusive behaviour of devices that are already in use. Therefore, we are looking to still derive useful knowledge from the less specific (non-intrusive) sensor network easily available at Smart Homes.

**Figure 1:**
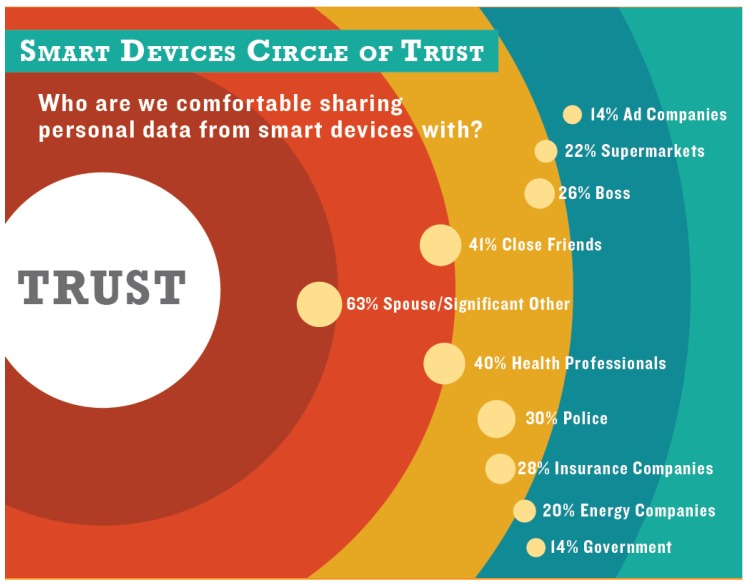
Circle of trust regarding the Internet of Things. Research carried out by TRUSTe. Source: [4].

According to the TRUSTe report [4], 83% of U.S. Internet users and 84% of British Internet users were concerned about the idea of information being collected through smart devices, and 87% of U.S. Internet users compared with 84% of British Internet users were concerned about the type of information collected by smart devices (see Figure 1). Although the IoT literature still lacks studies on the behavioural aspect that explains the customers’ perception of IoT adoption and focuses more on technological aspects, an IoT technology trust model is proposed in [5] that covers a varied set of factors related to consumer trust, which affects the decision to adopt IoT technology, including three main dimensions and eight domains. The three dimensions are: security of products and service-related factors, product-related factors, and social-influence-related factors. According to their survey analysis, the security and privacy of IoT products and services are among the highest priorities to ensure consumers’ trust; however, they remain a challenge in IoT technology. For this reason, this paper has the goal of promoting the development of useful behavioural models by learning from non-intrusive general-purpose sensors that are already in place to cover other functions. Additionally, because of the intrinsic uncertainties, it becomes relevant to analyse to what extent the uncertainty in the underlying datasets impacts the quality of the models.

A non-invasive environment for tracking the behaviour of individuals is particularly useful for the living environments of the ageing population, although some concerns regarding usability and user friendliness have been raised [6]. There is a wide range of solutions for monitoring the mobility of this population, but many proposals are based on the installation of new dedicated sensors [7]. However, it is also possible to take advantage of the already-existing infrastructure, designed mainly for other purposes, to infer information associated with people’s behaviour [6,8,9].

According to the World Health Organisation (WHO), over 47 million people live with dementia worldwide [10]. These figures are increasing, and, by 2050, the prevalence of Alzheimer’s-related diseases will quadruple [11]. Unusual behaviour and mobility patterns in the older population may be early symptoms of dementia [12]. The detection of these patterns would help provide a better intervention regarding the living environments of people in the early stages of dementia to improve both their well-being and that of their caregivers, and could help to delay institutionalisation [13]. On the other hand, analysing the behavioural changes in the routines of already-diagnosed people helps to evaluate their long-term changes and enables the implementation of effective assistance and palliative care [14]. Although specialised equipment and expertise are needed for accurate diagnosis, smart environments with ambient intelligence may raise the alarm and allow for further investigation to be made.

From the previous analysis different cohorts of population can benefit from soft monitoring systems able to analyse trends in different temporal scale, including elaborated information from the existing sensors. Such cohorts include risk attention to daily activities in the kitchen, initial steps of some diseases, and many other regular cases.

To show that these goals can be effectively reached, the remaining structure of the paper develops as follows. Section 2 reviews different relevant contributions in the field. Section 3 presents the promoted framework enabling the development of user-centred AmI applications and it defines the real context used to learn from behaviours. Later, in Section 4, the modelling effort is coherently developed for the context defined in the previous section. In Section 5, the results for the modelling activities are presented. Then, Section 6 discusses the results found in relation to the expectations, and it derives the impact of noise effects. Finally, Section 7 summarises the findings, establishes limitations and points out related topics suitable for further research.

## 2. Related Work Review

Older people want to remain independent while they have no disabilities that prevent them from doing so, or while their level of disability is manageable. In the U.S. alone, about 80% of those over 65 are living with at least one chronic disease, and an estimated 5.4 million senior citizens are suffering from Alzheimer’s disease [15]. To this end, AmI is a very useful tool, as it enables people to continue to live in their homes with sufficient confidence and security. Regarding the AmI, there are some interesting contributions, such as a theoretical one looking to establish an ontology between the two dimensions of AmI and smart buildings [16]. In other cases, the contribution is very narrow, and the studies emphasise a particular aspect, as in [17], which aims to control an LED system for lighting under more user-centred criteria, or the work in [18], which promotes learning from user behaviour to reduce energy usage.

Sometimes the AmI proposal involves specific components, such as a fuzzy embedded agent-based approach for realising AmI in intelligent inhabited environments for controlling the heating, ventilation and air conditioning (HVAC) subsystem of the smart building [19]. In other cases, the contribution is much more referential and it proposes a new way of understanding the phenomena, such as the proposal of a framework aiming to coordinate a set of agents for the management of the energy in a smart building [20]. There are also contributions that are very specific with regard to comparing features based on the IoT with people’s behaviour. One example is found in [21], which is concentrated on devices that measure by actigraphy instead of polysomnography and is looking to identify similarities between people with regard to their movements during periods of sleep. It is focused on a specific ethnographic community of people, and, therefore, could face issues to do with privacy.

There are also several interesting surveys, such as the one in [22], which presents its findings under five groups: privacy and security, sensing, reasoning, acting and human–computer interaction. There are some others, such as the one in [23], which emphasises the relationship between AmI and artificial intelligence (AI) to help identify and learn about user profiles, recognizing recurrent users’ behaviours, etc. Unfortunately, almost all the mentioned contributions were related to rather simplistic configurations with high levels of intrusive screening, including charge-coupled device (CCD)-based tracking, etc. Indeed, there are authors that seek to identify the most convenient tools, which emerged from the literature review as the most suitable and widespread, by comparing them to identify the interaction mechanisms that end users appreciate most [24]. To this end, there are also relevant contributions coming from ontology fields looking to provide insights to different contexts, such as hotels [25], etc.

Some researchers have identified smart homes as instances of AAL technologies. They have suggested that these AAL technologies can facilitate care, and consequently improve the quality of service provided to users [26]. Almost all the works in this field are based on the principle of event-condition-action (ECA), mainly due to the use of user interfaces for triggering actions from a program. AAL systems must represent many different types of contextual information, such as sensor information, activity structure, user profiles, temporal information such as environmental data, and spatial information such as the residence layout and its surroundings [26]. In this paper, the relevant AAL areas are identified as activity recognition, context modelling, anomaly detection, location and identification, and planning. For instance, planning makes it possible to schedule daily plans and to create flexible daily reminders to help dementia patients to carry out their daily activities [27]. Planning and scheduling techniques can be categorised into classical approaches such as forward and backward searches or graph-based analysis, decision-theoretic techniques such as Markov decision processes, hierarchical techniques, as well as those that are suggested by rule-based systems.

It is worth highlighting the contribution of Hao et al. [28], who focused on activity recognition as one of the most important prerequisites for smart home applications. They clearly identified that it is a challenging topic due to the high requirements for reliable data acquisition and efficient data analysis, which also emphasises the noise issue we have already highlighted. The critical factors that were identified as aggravating the complexity of recognition are the heterogeneous layouts of smart homes, the number of residents, and varied human behavioural patterns. Therefore, most human activity recognition systems are based on an unrealistic assumption that there is only one resident who is performing all the activities.

Most of the contributions to anomaly activity detection have been used for detecting anomalies in daily activities or medication compliance, by using rule-based techniques, similarity-based techniques or temporal relation discovery techniques. There was no transfer of knowledge involved as the learning activity recognition came from the same context. Therefore, learning in real noisy environments, identifying significant patterns and looking not just to fire rules to drive actions, but to increase the existing knowledge by using a general-purpose non-invasive sensing strategy could be interesting. It can be valuable to infer long-term bias with some degree of anticipation. To the best of our knowledge, this approach has not been analysed in depth previously.

Regarding the detection of human activity indoors, several approaches are currently being studied. Regarding human presence/movement detection, three main technologies are the current focus of analysis in the literature: environmental data-fusion, image/video and Wi-Fi/Bluetooth. Image-video-based solutions have been stated to obtain high-accuracy results; however, they suffer from privacy-related issues. Applications of this kind are presented in [29], where image-based detection is used to determine the occupancy of various university buildings. Wireless communication signals are also being used in two different ways. Firstly, they are used to count users with connected devices by analysing the footprints of the detected devices, as in [30]. Secondly, Wi-Fi signal perturbations are being used to infer presence and movement, as stated in [31,32]. Finally, environmental data-fusion refers to the use of environmental values such as temperature, relative humidity, CO${}_{2}$, etc., to infer additional information by using data-fusion and other data mining techniques, as shown in [33,34].

AAL technologies have also been studied to develop alarm-based monitoring systems to notify users of non-ordinary actions and behaviours in older people and people with physical disabilities. In the Italian project HDOMO, they developed an automated domestic environment that made it possible for people to continue being autonomous in their private environment. The behaviour of home inhabitants was monitored by using low-cost passive sensors alongside depth cameras. They developed a series of alarms that detected unusual behaviours such as falls, sleep apnoea, incorrect feeding, etc. [35]. Other approaches, such as the one presented in [36], define systems that learn from room-to-room transitions and permanence to detect behaviour changes that may be related with health problems, which are communicated via alarms. Apart from sensors installed in built environments, the application of wearable devices has also been studied to detect attention-requiring actions (e.g., low blood pressure), enabling monitoring to continue outside homes [37].

Based on the findings from the literature review, the authors agreed with the the major limitations reported, which are that recognition activities are based on unrealistic hypotheses, and mainly used in highly monitored environments that are difficult to generalise. Therefore, this study selected a multi-human environment with different behavioural patterns as the source for learning user practices (see Section 3). Indeed, we suggest the idea of using AI techniques to deal with non-intrusive and non-human dedicated sensing activities to contribute to certain human activity recognition, when some context exists which provides insight for location identification in order to make it possible to use transfer learning by applying classification pattern analysis to help in anomaly detection for other contexts. In the next section, more details are provided regarding the selected context.

After analysis of the contributions, we can identify a gap related to the lack of consideration of identification of behavioural rules people adopt when different time scales are involved. Our vision is that, because such rules involve created knowledge, bias from the regular behaviour of people can be identified, and, by understanding their significance, potential negative effects or risk can be mitigated. This is of crucial importance in the case of ageing people living alone, as it can lead to early warning of potential approaching difficulties such as memory loss, unsafe usage of facilities, etc. Therefore, this paper aims to contribute to the research in this field.

## 3. Materials and Methods

To evaluate the potential of such rule discovering capabilities, we selected a convenient context. The research environment was carried out in a care home for ageing residents with dementia who are in long-term care.

The Technology Acceptance questionnaire was not administered as such, because the non-invasive design for the experience and the characteristics of the residents. Instead, an informed consent was given by each participant or a responsible family member stating that only anonymous data would be used for epidemiological, public health, educational or research purposes.

**Figure 2:**
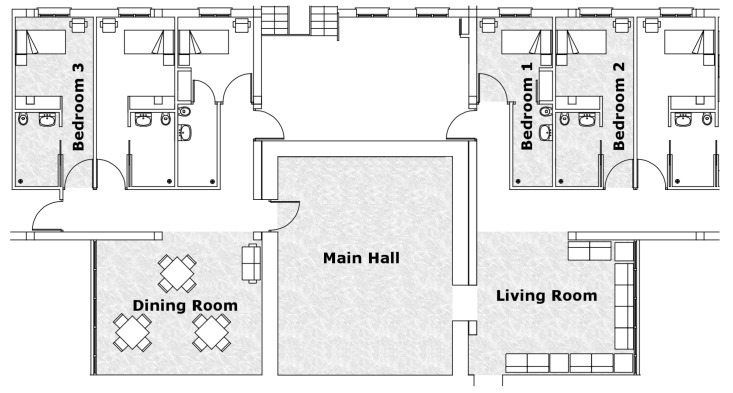
Area of interest: A cohabitation unit in the psychogeriatric ward of an elderly care home.

The care home allowed us to install and monitor non-intrusive sensors in six different rooms of the psychogeriatric ward located in the second floor of the building: the Main Hall, the living room, the dining room, and three bedrooms spread over the same independent cohabitation unit. The three monitored rooms were chosen because the residents with major behaviour problems resided there and, therefore, they were in need of more assistance and attention. Among the incidences that sometimes occur are agitation, alteration, screams, verbal aggression, physical aggression and inappropriate behaviour. The site layout plan with the monitored rooms depicted on a grey background is shown in Figure 2.

Thirteen residents were living in the cohabitation unit during the year in which data were collected. The residents were seven men and six women with an average age of 84 years. A predetermined timetable of the care home provided clues about the residents’ normal location, making abnormalities stand out more clearly. Residents were in bed between 22:00 and 08:00. From 8:00 on, residents were woken up, cleaned and dressed by the nursing assistants. They spent most of the day between the Main Hall and the living room, connected by a narrow passage. They usually only accessed the Dining Room during meals (8:30–10:30, 13:00–14:00 and 19:00–20:00). Bedrooms were occupied at night, although they may have occasionally been used during the day (e.g., nap after lunch), and since residents can receive visits from relatives and the bedrooms closest to the living room are not locked. Almost half of the residents need to be moved from one space to another because they do not move on their own. On the other hand, other residents roam freely.

The six monitored rooms were equipped with identical wireless sensors to measure and report levels of ambient temperature (${}^{\circ }$C), relative humidity RH (%), CO${}_{2}$ concentration (ppm) and passive infrared (PIR) technology-based motion detection. The temperature, relative humidity and CO${}_{2}$ levels were collected by multisensor devices mounted on the wall of each room in the “breathing zone”. The “breathing zone” was located at a height of 1.5 m above the floor in the Main Hall, the living room and the dining room, and at a height of 1.2 m, next to the bedside tables, in the bedrooms. The PIR sensors were mounted on the ceiling, in the middle of the shared lounges, and above the bed area in the bedrooms. These sensors were primarily designed for the control of lighting, heating, ventilation, and air conditioning systems (HVAC) and characterised as being easily installable and non-invasive. During normal operation, the sensors harnessed light energy from the environment through integrated solar cells and transmitted the measured data to a central node that uploaded them into the cloud. At night or in low light, backup batteries provided additional power to ensure the proper operation of the sensors. All sensors support the EnOcean™ standard protocol (ISO/IEC 14543-3-10) for low-power wireless communications.

The data acquisition was carried out with respect to the national laws regarding non-intrusiveness, privacy and confidentiality over the period of one year. The multisensor devices collected environmental data regarding the temperature, relative humidity and CO${}_{2}$ levels at a regular frequency that could decrease during extended periods of low light due to low battery charge. However, the ceiling-mounted PIR motion sensors operated as room occupancy gauges by detecting when a room was occupied or vacant. These PIR sensors did not send the motion detection data periodically but when the room occupation status changed with minimum pre-configured delays. In this way, when motion was detected, the data indicating the occupied statuses were transmitted immediately and no more data were sent in the 2 min that followed. Then, the PIR sensor transmitted an occupancy message upon detection of a new motion. On the contrary, if no motion was detected for an extended period of 10 min, an unoccupied message was sent. These pre-configured delays optimised the energy consumption of the PIR sensors without affecting the effective control of the HVAC and lighting systems; however, they made data analysis more challenging as no information regarding the room occupancy was sent between delays. Therefore, the sampling frequency of the environmental sensors was finally established to be 10 min because that frequency matched the restrictions of the PIR sensors, that do not send an unoccupied message until ten minutes have elapsed since the last motion detection. In addition, the power generation requirements under normal lighting conditions do not allow the frequency to be much higher than ten minutes and indoor environmental values usually remain stable without sudden changes.

The installed sensors provided information on the quality of the environmental parameters in which residents lived, as well as information regarding their movements. A deeper analysis to discover behaviour and mobility patterns of the residents and the integration of additional information, such as the timetable of the care home, could reveal aspects associated with the evolution of the disease. In general, the identification of either sudden or progressive changes in their routines helps to provide specific care to the residents.

The data collection provided more than 50,000 patterns, considering the raw sensor data. Careful labelling process was carried out for 16,900 patterns to be used for the presence data model, and about 560 patterns for the window opening process, as it is a less frequent event and limited unbalanced datasets were used. The patterns used for the presence model were randomly segmented into 11,323 for training and 5577 for testing. In a similar way, the 560 patterns for the opening window model were segmented into 392 for training and 168 for testing. It became clear that specific effort was required for labelling the patterns, therefore it was indicated that the chosen case study had a partially labelled dataset.

After the description of the site, the sensing strategy adopted, and the data collection carried out, the method used for data preparation is presented here. Note that we were interested in developing software-based sensors that were able to determine and formalise new knowledge. Two different models were proposed, the first one was regarding the presence or absence of people inside a room during a significant period of time, only taking into account the patterns of CO${}_{2}$, temperature and humidity evolution. The second model was to detect when the window in the room was opened or closed. As described above, the environment was very noisy, not only because of the occasional failure of the sensors but due to additional unforeseen behaviours such as nurses leaving doors opened, forced ventilation rules, diffusion of air (as well as the related physical properties) between spaces, which all occurred without any clear pattern.

To be able to support the conclusions found in an unbiased context, data preparation adopted the removal of sequences of days from the datasets for learning purposes. These removed data became the elements that were used to test the knowledge gathered after the training processes had ended. For the remaining data in the datasets, as several configurations required testing, a cross-validation folder approach was adopted to decide the most suitable modelling configuration.

Indeed, regarding the context awareness discussed in the state of the art AAL approaches, it was decided to incorporate some degree of semantic description of the spaces, making it possible to be responsive not only to the indoor variations of parameters, but also to those from the next room, thus adding information about the building. The usage of Web Ontology technology enables to setup a clear relationship between the sensor data and the home context towards defining the activity context. It enables not only the description of the entities themselves, but also their relationships in a formal way suitable for being exploited in the model construction [38]. Indeed, services, home owners but also family relatives can be easily defined, as well as time references. Based on the established relationships both geometric but also functional properties can be named. Geometric properties can help in improving the modelling task but functional are in the basis for the rule adoption, as depicted in the Discussion Section.

## 4. Modelling

The first aspect to be analysed is what is known in the cross-industry standard process for data mining (CRISP-DM) methodology as “data understanding”, in the context of the business understanding. To this end, some pictures of the variables per monitored space based on the expected seasonality, which is a daily behaviour, can help to understand the particularities. From the analysis, it became evident that the relative position of the sensors for the same type of spaces (e.g., bedrooms) was significant in terms of the absolute values. Indeed, the geographical orientation of the spaces inside the building also became relevant, as the relative humidity (RH) and temperature levels were significantly different for different orientations when the bedrooms were considered.

Presence sensors provide very noisy information, as their operational design makes the movement signal last for a long period. On the contrary, if people do not move at all, the sensors indicate the absence of people, which is not true. Indeed, the movements of nurses and other working people contribute to their activation. In the end, and after various filtering strategies, it was decided that the PIR information was mainly useless because of its high noise level.

However, those sensors allowed us to appreciate when ventilation occurs during room cleaning in the morning as RH spiked and temperature also became impacted. Additionally, a reduction of CO${}_{2}$ was seen when users left a room.

Overall, it was evident that RH and temperature were strongly regulated by the building control system and they were, therefore, not very informative. However, the CO${}_{2}$ sensors were still useful as they provide information regarding the room occupancy. The basic principle of its behaviour is that the CO${}_{2}$ concentration increases over time, as the person sleeping inside is continuously producing it. It is also expected that when the room becomes empty its concentration will be lower than when it is in use. Obviously, real behaviour is slightly more complex because sometimes the door to the room remains open when the room’s user is inside and diffusion happens, which moderates or reverses the growth in CO${}_{2}$ concentration.

**Figure 3:**
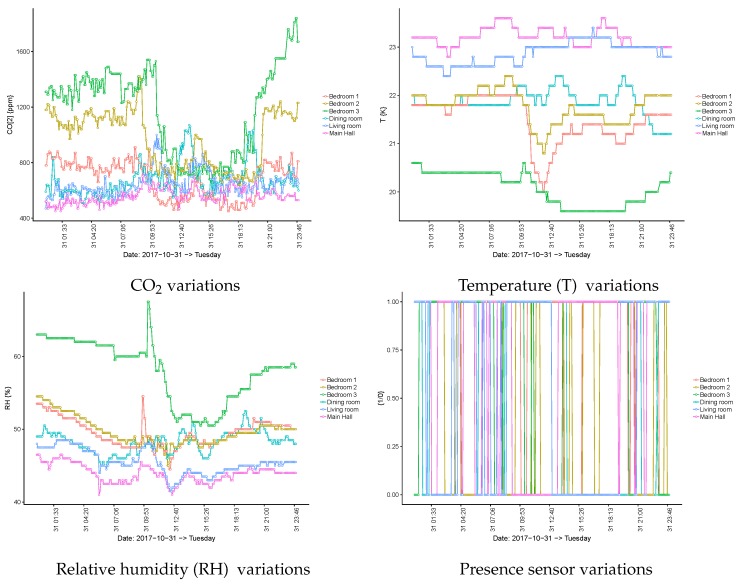
Sensor variations per room on 31 October 2017.

**Figure 4:**
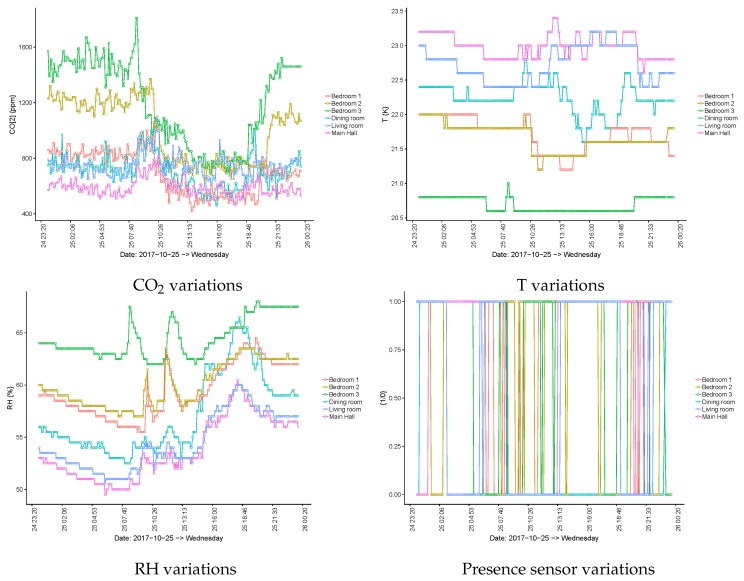
Sensor variations per room on 25 October 2018.

By inspecting Figure 3, it can be seen that the users of bedrooms 1 and 2 spent some time resting after lunch and that this was not the case for the user of bedroom 3. The figure also shows that, in bedroom 3, the CO${}_{2}$ level began to increase after dinner time (20:30), and during dinner, the CO${}_{2}$ flowed from the dining room to bedroom 3 when the room’s door remained open due to its proximity. These effects are relevant and reinforce the need for the semantic description of the building. By observing the same levels during night time, it becomes clear that the CO${}_{2}$ concentration does not increase in bedrooms (e.g., bedroom 2) in the first part of the night, whereas some increases occur in the living room and in the main hall. This behaviour changes around 07:00 when the nurse visits the room and the bedroom’s door is closed. Then, CO${}_{2}$ starts to increase again. If the focus becomes the dining room, it is possible to identify breakfast time (short period of time around 08:30), lunch time (larger period of time around 13:00), and dinner time (at around 19:00). The Figure 4 shows a rather similar behaviour for the same spaces, but in a different point in time.

The previous analysis makes it evident that some knowledge can be derived from the sensors’ data. Mainly, the rules that were found were related to the room occupancy for users, where the aggregation of data from sensors over time must be significant in order to deal with the previously highlighted local variations.

Therefore, we were interested in building models that inferred the use of the spaces, by considering the level of CO${}_{2}$, the slope of its variation, the values from the neighbouring rooms, and the RH variations. To adequately learn from the data, a smooth data function was required, and therefore a moving average of five values was used.

Then, a model for presence in bedrooms was trained by preparing a dataset that involved the following variables:

- CO${}_{2}$ concentration level in the room.
- CO${}_{2}$ concentration level in the contiguous room.
- Slope of the regression linear model for RH level in the room considering three points before the present time and three points after.
- Slope of the regression linear model for CO${}_{2}$ level in the room considering three points before the present time and three points after.
- Slope of the regression linear model for CO${}_{2}$ level in the contiguous room considering three points before the present time and three points after.

Labels for the outcome variable were adopted according to the reported information available from the care home.

For the model related to the window status, the variables under consideration were:

- CO${}_{2}$ concentration level in the room.
- Relative humidity in the room (RH).
- Slope of the regression linear model for RH level in the room considering three points before the present time and three points after.
- Slope of the regression linear model for CO${}_{2}$ level in the room considering three points before the present time and three points after.

As already indicated in the last part of the Materials and Methods Section, to preserve the independence for validating the learned model, the information from January 2018, after the 10 January 2018, was removed from the dataset and reserved for testing purposes. The remaining part of the dataset that was used for learning purposes involved 11,323 samples with an unbalanced ratio between classes of 66%/33% (i.e., presence/absence from the bedroom).

The goal when addressing artificial intelligence techniques is not just to find models that can produce accurate predictions, but also can be used to extract knowledge in an intelligible way, i.e., can be understood as being able to explain the reasoning logic behind the provided outcome.

Although there are many techniques and algorithms helpful for this purpose, tree-based methods stand as one of the most effective and useful method, capable to produce both reliable and understandable results, on mostly any kind of data [39]. Therefore, although different methods were explored such as naive Bayes, support vector machine and feed forward back propagation neural networks, the final decision was to use random forest technique, as a good representative technique for the tree based classification algorithms. As a summary for their benefits are:

- Decision trees are non-parametric. They can model arbitrarily complex relations between inputs and outputs, without any a priori assumption. Indeed, these methods do not require variable standardisation and other transformations, which can hinder future usage as values can go out of the adopted range.
- Decision trees handle heterogeneous data (ordered or categorical variables, or a mix of both). This become an interesting characteristic for classification problems.
- Decision trees intrinsically implement feature selection, making them robust to irrelevant or noisy variables (at least to some extent). Actually, it is a convenient characteristic that was used in this study to identify the most contributing variables. Indeed, they help to understand the path for the provided outcome.
- Decision trees are robust to outliers or errors in labels. This characteristic becomes critical in real life applications, when sensors can have issues and provide either wrong or biased measures.

Therefore, the adopted modelling approach was the random forest classifier [40] because of its flexibility, ensemble capabilities, and bagging properties. During the tuning process, different parameters were tested, such as the number of variables considered for the trees (between two and five were analysed and two was the value finally adopted), the number of trees considered for the ensemble (between 100 and 600, where 500 was selected) and a strategy of a 10-fold cross-validation was adopted to avoid bias due to specific pattern selection for training. The same strategy was used for both models and the adopted parameters were the same.

Creating a classification and regression tree (CART), involves selecting input variables and split points on those variables until a suitable tree is constructed. The selection of which input variable to use and the specific split or cut-point is chosen using a greedy algorithm to minimise a cost function. It is a recursive binary splitting and tree construction ends using a predefined stopping criterion, such as a minimum number of training instances assigned to each leaf node of the tree.

The random forest technique relys on the consensus carried out by different trees (the forest). When the training set for the current tree is drawn by sampling with replacement, about one-third of the cases are left out of the sample. Each tree is constructed using a different bootstrap sample from the original data. About one-third of the cases are left out of the bootstrap sample and not used in the construction of the *k*th tree. These out-of-bag data are used to get a running unbiased estimate of the classification error as trees are added to the forest. It is also used to get estimates of variable importance.

In random forests, there is no need for cross-validation or a separate test set to get an unbiased estimate of the test set error. It is estimated internally, during the run of the algorithm, which makes it even less dependent on the configuration decisions.

After each tree is built, all of the data are run down the tree, and proximities are computed for each pair of cases. If two cases occupy the same terminal node, their proximity is increased by one. At the end of the run, the proximities are normalized by dividing by the number of trees, and they become useful in replacing missing data and locating outliers [40].

The objective here is not to develop a specific study regarding the capabilities of machine learning to represent the knowledge but to use almost-standard and sufficiently robust techniques to capture and properly represent this new knowledge. The new knowledge distilled is represented by the two models built, and as far as such information was not measured but just inferred by the models from the general purpose sensors, they are seen as soft sensors. This soft sensors become key elements regarding different problems, as part of the assessment for the construction of the behavioural rule.

## 5. Results

The two selected models were trialled under a strategy of 10-fold cross-validation with minimal adjustments for the selected variables as per the split of the standard recursive partitioning algorithm used, which starts with all the data and performs an exhaustive search looking for possible split points to find the one that best explains the entire data, or, in formal terms, that reduces the node impurity the most. Cross-validation ensures that the averaged performance being considered is independent from the set of data.

The results show that, for this particular case, it was best to only consider two variables, as depicted in Table 1, where the accuracy represents the percentage of correctly classified instances out of all the instances, and Cohen’s Kappa essentially depicts the classification accuracy, except it is normalised at the baseline of random chance on the dataset.

Validation of the trained models was carried out using data from the same room but measured during a period after the one used for training. Therefore, full independence of the data was adopted and the results are presented in Table 2.

From the results in this table, obtained from data not previously seen as the data came from the part of the dataset that was omitted for validation, it becomes clear that the accuracy of the classifier was high (F1-score: 0.984).

After validating the coherence of the model, it was then necessary to determine which variables the created knowledge was based upon, which is presented in Table 3.

To this end, it becomes clear that the presence model has a high accuracy level, and the highest impact for the decision-making process regarding the presence/absence of the user in the bedroom was the CO${}_{2}$ concentration (with a significant contribution from the variation rate (i.e., the slope) of this concentration). There were some other relevant effects we were unable to consider, such as the effective contribution of the forced ventilation system with regard to the variation in bedroom temperature, etc., as such data were not accessible. The window model was also considered and the results are presented in Table 4.

For this model, the accuracy metrics (F1-score) was equal to 0.935 and it was considered acceptable. Indeed, it was possible to understand upon which variables the knowledge created was based, which is presented in Table 5.

The built models allowed for additional knowledge by means of the new features to be obtained. These features, which are not directly measured by the existing sensors, allow for estimation of the room occupancy time and the ventilation routine of the room to be carried out on a daily basis. Indeed, in Table 6, the models were applied to the data collected during several days and the new information was obtained.

**Figure 5:**
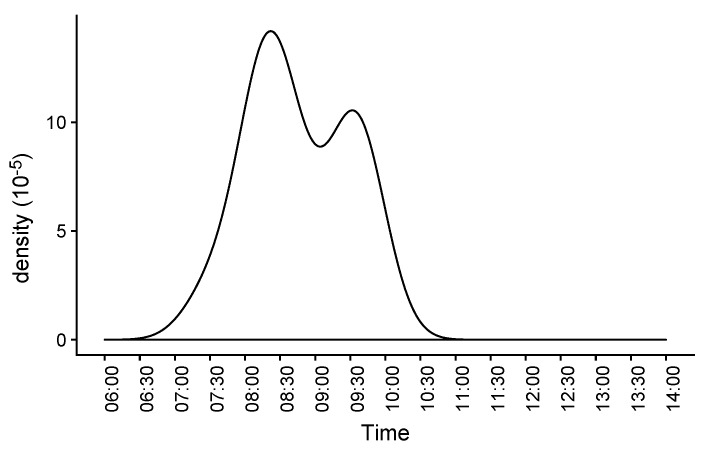
Frequency of room ventilation based on the opening of the window.

Based on the information obtained from the models, it becomes possible to estimate frequent behaviours, such as the routine for ventilation based on the time of opening the window in the room (see Figure 5).

**Figure 6:**
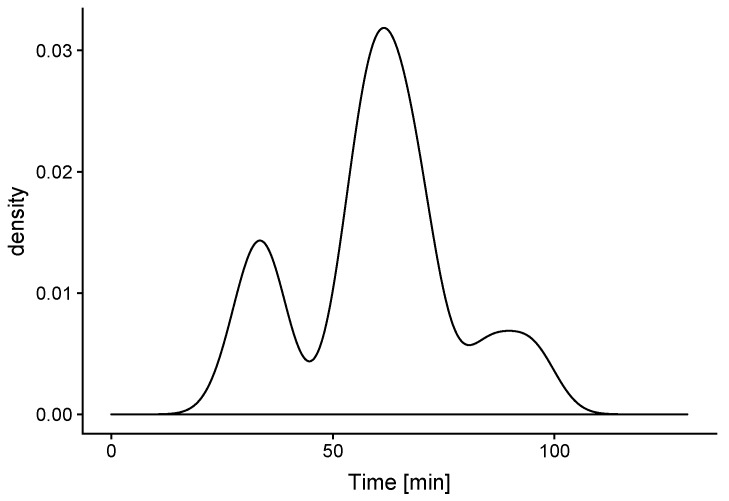
Duration of ventilation activities.

Furthermore, it can be estimated how long such process lasted, as shown in Figure 6.

**Figure 7:**
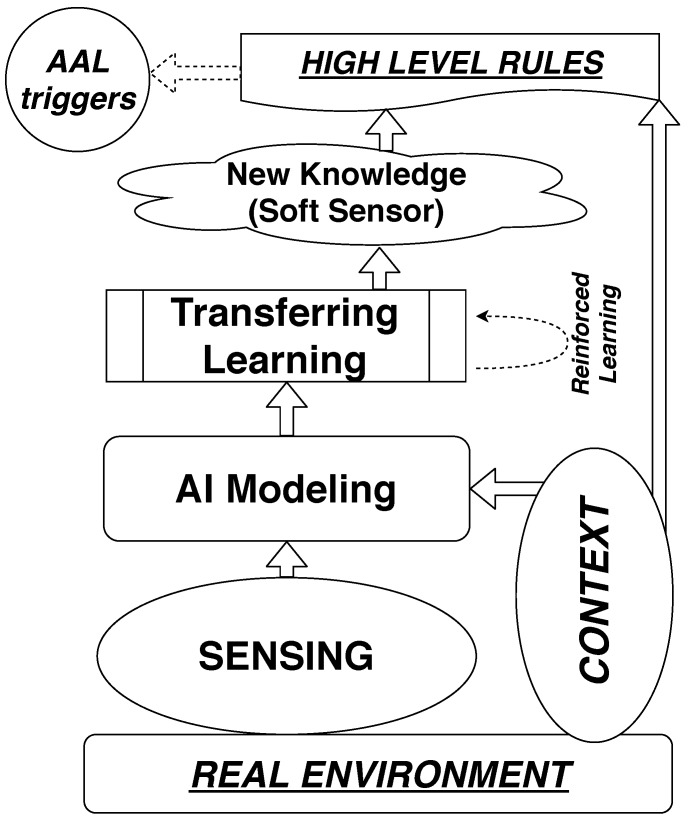
Referential framework proposed. AAL, ambient assisted living; AI, artificial intelligence.

This information can be refined with regard to a specific period such as in summer or in winter, or for working days and weekends. Therefore, behaviour can be compared against that which was exhibited during the preceding days and it can be determined when it becomes unexpected. For instance, one rule could read as follows, “if during last three days the ventilation last more than the maximum threshold then raise an alert” or “if more than three times during last ten days there are absences from the room lasting more than 10 min each between 22:00 and 07:00, then alert”. This is because such behaviours are understood as potential signals for behavioural changes that needed to be analysed. All such rules can be implemented in the reasoning layer, named “High Level Rules” according to the proposed framework in Figure 7.

To provide a consistent context, capable of managing the structure proposed in the depicted framework, both for handling the complexity of different rules, and their updating process, and for enabling the regular accuracy monitoring of the built soft sensors and launched the reinforced learning processes when required, it is proposed to adopt the usage of Web Ontology Language (OWL), in a particular semantic context expression, given the basis of satisfying context-aware reasoning. Particular attention is deserved to the rules designed with Semantic Web Rule Language (SWRL) [38].

The approach will typically enable the implementation of effective strategies to help in the management of different use cases in a flexible way, as the resulting ontologies are essentially shared knowledge models that enhance the capabilities of automated processing and the level of automation by allowing machines or agents to interpret data/information and reason against ontological contexts, thus enabling knowledge based intelligent decision support [41]. Applications have also been reported in the context of IoT, as in [9], where similar architecture was proposed for Cruise cabins.

## 6. Discussion

As explained in the previous sections, the a priori effect of a human presence on the temperature and humidity of a room is limited by artificial regulation systems. However, we were able to detect window openings in the form of high immediate increases in humidity, which of course were influenced by the climate in the monitored zone where normal humidity values were between 70% and 80%.

In contrast with temperature and humidity, CO${}_{2}$ values were shown to be highly correlated with human presence and, that being the case, this was the parameter used to determine room occupancy in this study. Not only were the on-site values used to determine human presence but measurements in nearby large spaces were also used, as they had a great effect on the contiguous rooms. This was especially important when the doors were opened and the diffusion of air between spaces was more notable. In this way, information regarding the physical distribution of the building was added to the models.

The aim of this work was to enable the detection of regular behaviours to aid in the monitoring of patterns, such as occupancy or ventilation, based on normal environmental sensors which are already present in many spaces for HVAC control, etc. When this purpose was achieved, the information gathered by these sensors could be modelled to gain additional information without adding more sensors or infrastructure to the building. To validate our proposal, additional sensors were installed in the form of PIR sensors to gather basic knowledge of binary presence (i.e., occupied/unoccupied) in the rooms. The aim here was to avoid installing these supplementary devices while being able to obtain the knowledge they provide. Indeed, this was a significant conclusion because the sensor does not regard presence but movement.

The viability of using machine learning models, in this particular case by using the random forest method, for this problem was demonstrated for different problems, obtaining high accuracy values (see Table 1 and Table 4), when a 10-fold cross-validation strategy was adopted.

All the possible configurations of the data that could be used in the models were tested, and, finally, the one that obtained the most accurate results was the model using trees with two variables in the forest. The trained model with just two variables was validated with previously unseen data, obtaining an accuracy score of 98% (the confusion matrix is shown in Table 2) for the room occupancy and 93% for the ventilation model. With the results obtained, the presented approach is considered to be valid to determine the presence in rooms in which the data were used to train the models. However, further work is needed to generate a unique model that could be transferred to other rooms.

Based on the knowledge gathered, it should be possible to transfer the knowledge obtained in one room to others, by using the model to estimate the presence of people in other bedrooms. However, because of the relevance established in Table 3 for the different types of variables, it becomes clear that absolute levels of CO${}_{2}$ have strong relevance. Therefore, the model cannot be attributed to specific rooms and it provided unacceptable failure rates when applied to different rooms.

When transferability becomes a requirement for the knowledge gathered, a different strategy must be adopted (e.g., variable normalisation), in order to make it possible to compare scenarios. The size of the rooms could also be needed for such normalisation, as the effect of one person’s presence does not affect a bedroom or the Main Hall in the same way due to their size difference. As this aspect is a relevant factor, specific tests were performed to analyse the effects when learning was transferred. To this end, a normalisation between 0 and 1 for the CO${}_{2}$ and RH values per room was performed (as effective relative volumes of rooms were not available). The same variables were defined, including the slopes of the normalised variables and the same type of random forest model was built, with exactly the same set of values for training and with the same strategy for validation. Therefore, the results can be interpreted using the same contingency table as Table 2 in comparison with the actual contingency table (see Table 7), and it was concluded that the quality was similar for both models.

Subsequently, it was possible to transfer the model to Bedroom 1. This was particularly significant, as the behaviour observed in Bedroom 1 was less defined than in the other bedrooms (see Figure 3). The contingency table describing the performance of the model is presented in Table 8.

Indeed, some improvements could be made when reinforcement learning was considered. However, even though the performance was slightly reduced, the value of transferring knowledge can still be easily appreciated. The F1 score as averaged value per class was reduced from 0.984 in the proper context up to 0.84 when transferring was considered.

It is also necessary to recognise that the use of these sorts of models requires a certain level of abstraction because, in the creation of the synthetic variables, a time summarising approach must be adopted. As previously commented, values within (t−30min,t+30min) were used to calculate the slopes for moment *t*. Therefore, the decision was made based on hourly behaviour instead of having a prediction at the same frequency as the sampling rates.

In addition to transferring models between contexts, which sometimes requires the application of reinforced learning from a small amount of data coming from the present room in addition to the already trained model in another space, an additional use can be derived from the created knowledge. This involves the establishment of rules by comparing the learned long-term behaviour with the present one. As an example, by comparing the starting routine for performing room ventilation (see Figure 5) with the recent and short-term (for instance last week) pattern, potential advice can be derived and, when combined with other factors, such as variations in the room usage, etc., they can alert against early effects that can require extra investigation. Another potential rule could also consider the ventilation time used in connection with the outdoor weather conditions. The criteria for raising alerts will depend on firing several of these rules at the same time, in order to collect stronger evidence for any change in behavioural patterns.

Through the studied case it becomes pretty clear that the quality of the derived knowledge strongly depends on the labelling processes required both for training at the beginning and for reinforced learning in the case of update of the behaviour or transference of the models to a different environment. Actually, this dependency becomes an opportunity to offer the people being monitored to participate and to be at the same time users but also co-creators of the Aml technology. If a convenient and simple phone app is enabled to ask the participants willing to adopt such active role, the regular labelling process can be enriched dramatically, and full set of benefits can be derived, at the system level but also at individual level. To foster such dynamics and provide a smart system just querying when relevant events happen, and also for making a consistent management of the produced rules possible, the assistance from the semantic perspective becomes essential. Therefore, the natural evolution for using such soft sensors is to integrate them into a semantic schema able to provide a holistic perspective.

## 7. Conclusions

The ability to determine human presence in the spaces of a building without adding additional sensors in a non-intrusive way could be applied to detect behaviours that are not understood to be regular in cases where regular patterns can be established, such as in the case of elderly people living alone or in cases such as the one described in this paper, where patients display very regular behaviour day-to-day. The acquired knowledge allows the authors to propose the reference framework depicted in Figure 7.

Either from the direct model outcome or by using transferring strategies, the newly created knowledge can be seen as a kind of aggregated soft sensor where rules can be derived, such as the number of hours per weekday the user sleeps or the number and duration of absence times during the night time per weekday. Such newly derived knowledge can be used to establish alerts or triggers. This is significant as these alerts can inform the user of any unexpected long-term deviation from regular behaviour (e.g., month-over-month). This kind of soft surveillance will help to identify mental diseases or certain other difficulties in their early stages. The applications of the proposed framework will be further analysed in future research.

Although limitations exist because of the sample sizes and the strict context needed for labelling the samples helping in producing the supervised learning, the research carried out still shows strong capabilities for building soft sensors, and their advantage to establish variations in a different time scale, which can be useful for higher level behavioural rules. To this end, integration with semantic schema need to be deeply investigated in future research, in particular the rule management and updating procedures according to the confidence estimations.

In addition, in terms of limitations, it is relevant to emphasise that there is a bias towards the benefits and the positive outcome of the AAL technologies in particular in the case of aging people. This field is named as gerontechnology [42]. In this sense, the present paper revolves on the same idea of becoming useful to the relatives and the older people as well. However, it is worth recognising the lack of enough social studies with an agnostic lens trying to identify negative impacts of AAL over such collective, either because of the opt-out or just because of disruptions because of unsuccessful integration among components or AAL programs. Indeed, derived effects such as increased levels of social isolation because of the technology adoption will require further attention.

## Figures and Tables

**Table 1:** Cross-validated performance by considering different numbers of variables regarding the presence model.

Number of Variables	Accuracy	Kappa
1	0.9935522	0.9851646
2	0.9963665	0.9916528
3	0.9960815	0.9909948
4	0.9961171	0.9910764
5	0.9954046	0.9894418

**Table 2:** Contingency table for the presence classifier.

	Real Room Absence	Real Room Presence
Predicted room absence	2510	18
Predicted room presence	86	2963

**Table 3:** Relevance of variables for the presence model. Gini coefficient is related to Mean Decrease in Impurity.

Variable	Absence (%)	Presence (%)	GINI Decrease
CO${}_{2}$ in Bedroom 2	31.25	38.47	4644.5
CO${}_{2}$ in Living Room	25.03	32.24	3467.6
RH slope	20.53	13.14	2627.8
CO${}_{2}$ slope in Bedroom	11.40	8.87	746.9
CO${}_{2}$ slope in Living Room	11.79	7.28	733.6

**Table 4:** Contingency table for the opened window classifier.

	Real Room Absence	Real Room Presence
Predicted room absence	97	8
Predicted room presence	5	58

**Table 5:** Relevance of variables for the window model.

Variable	Closed (%)	Opened (%)	GINI Decrease
CO${}_{2}$ in Bedroom	58.79	48.55	73.44
RH in Bedroom	50.67	54.98	69.06
RH slope	55.75	48.47	85.07
CO${}_{2}$ slope in Bedroom	63.22	50.96	100.75

**Table 6:** New knowledge creation for bedoom 1.

Date	Inside from	Inside Until	Opened Window
26 January 2018	20:35	08:05	08:10/09:15
27 January 2018	21:05	08:15	08:20/09:05
…	…	…	…

**Table 7:** Contingency table for the normalised classifier.

	Real Room Absence	Real Room Presence
Predicted room absence	2517	18
Predicted room presence	79	2963

**Table 8:** Contingency table for the “transferred” normalised classifier.

	Real Room Absence	Real Room Presence
Predicted room absence	9976	774
Predicted room presence	468	16279

## Abbreviations

The following abbreviations are used in this manuscript:

AmI	Ambient Intelligence
AAL	Ambient Assisted Living
IoT	Internet of Things
HVAC	Heating, Ventilation and Air Conditioning
AI	Artificial Intelligence
RH	Relative Humidity
